# Silver Nanoparticles in Antibacterial Research: Mechanisms, Applications, and Emerging Perspectives

**DOI:** 10.3390/ijms27020927

**Published:** 2026-01-16

**Authors:** Hasan Karataş, Furkan Eker, Emir Akdaşçi, Mikhael Bechelany, Sercan Karav

**Affiliations:** 1 Department of Molecular Biology and Genetics, Çanakkale Onsekiz Mart University, Çanakkale 17100, Türkiye; hasankaratass2005@gmail.com (H.K.); furkan.eker@stu.comu.edu.tr (F.E.); emirakdasci@stu.comu.edu.tr (E.A.); 2 European Institute for Membranes (IEM)—UMR 5635, University of Montpellier, École Nationale Supérieure de Chimie de Montpellier (ENSCM), Centre National de la Recherche Scientifique (CNRS), 34090 Montpellier, France

**Keywords:** silver nanoparticles, antibacterial activity, agricultural applications, biomedical applications, toxicity

## Abstract

Silver nanoparticles (AgNPs) possess distinct physicochemical characteristics and demonstrate high antibacterial potential that highlights them as promising alternatives against a wide range of pathogens. The immense antibacterial potential of AgNPs is primarily attributed to the release of silver ions that lead to the disruption of bacterial cell membrane, generation of reactive oxygen species (ROS), inhibition of protein synthesis and interference with DNA replication. Variations in AgNPs’ shape, size, and surface characteristics are also considered key factors determining their effectivity as well as specificity. AgNPs are considered potent antibacterial agents, including against antibiotic- and drug-resistant strains. However, inappropriate dosages or unoptimized application of may result in potential toxicity, consisting one of the main drawbacks of the AgNPs’ safer administration. This article reviews the recent literature on the antibacterial potential of AgNPs, focusing on their broad mechanisms of action, applicability, especially in agriculture, biomedical and environmental fields, toxicity and future perspectives.

## 1. Introduction

Nanoparticles (NPs) are one of the most studied nanomaterials in the field of nanotechnology, typically within the size range from 1 to 100 nm with a size scale comparable to other nanoscale materials such as nanoclusters. At this scale, NPs exhibit unique chemical, physical, and biological properties that distinguish them from bulk materials. AgNPs can be classified into different categories depending on their physicochemical characteristics, such as size, shape, and surface charge. These properties are highly dependent on the bulk material which they have synthesized, including silver, gold or carbon-based materials. Lately, NPs are extensively investigated due to these diverse features. Among them, AgNPs particularly stand out with their broad range of distinctive properties [1].

One of the significant threats to public health today is the rise in pathogens that are resistant to multiple drugs. These drug-resistant pathogens are often at the forefront of infections [2]. Currently, researchers are actively seeking for new approaches to overcome the strong resistance of these pathogens. One of these approaches include the utilization of NPs, especially the AgNPs. Numerous studies [3,4,5] have shown that AgNPs are highly effective in inhibiting multidrug-resistant bacterial pathogens. In addition, although AgNPs exhibit strong antibacterial activity on their own, they are also frequently used in combination with conventional antibiotics to restore or enhance antibiotic efficacy. Such synergistic interaction can significantly amplify the antibacterial effects of various antibiotics, resulting in improved therapeutic outcomes [6].

AgNPs can inhibit diverse pathogens with multiple mechanisms of actions, promoting their applicability in antibacterial research. Some of these mechanisms can act simultaneously, which increases the effectiveness of the antibacterial activity. AgNPs’ interaction with the bacterial cell wall consists of one of the main mechanisms of their antibacterial activity. Such interactions lead to notable structural changes on the cell wall, causing significant membrane damage [7]. Similarly, AgNPs induce high levels of ROS synthesis that is related to most of the antibacterial mechanisms. The excessive intracellular ROS levels cause high oxidative stress, damaging essential biomolecules and disrupting the composition of the bacteria [8]. Additionally, Ag^+^ ions released from AgNPs can penetrate the cell membrane and interact with intracellular targets, further hindering the bacteria to function [9,10]. Furthermore, interaction between AgNPs and DNA and ribosomes can damage the genetic material, preventing replication and protein synthesis which is essential to the bacteria [11]. Despite these multifunctional mechanisms, the specific mechanism behind the antibacterial activity is not precisely explained [12].

**Figure 1:**
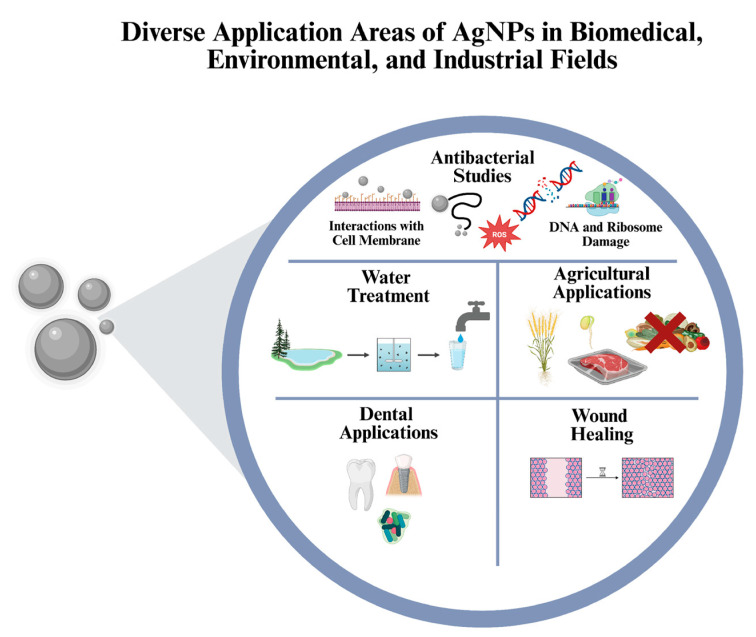
Utilization of Silver Nanoparticles in Diverse Fields. AgNPs exhibit antibacterial activity through interactions with microbial cell membranes, DNA, and ribosomes. They are widely applied in water treatment, agriculture, dental materials, and wound healing. The schematic illustrates these major application areas, where arrows indicate process flows or interaction pathways, and red symbols denote antimicrobial inhibition and oxidative stress generation [23].

Thanks to their strong antibacterial activity, AgNPs are widely utilized in various fields [13]. Their antibacterial applications are extensive (Figure 1), and numerous studies have investigated their effects on different pathogenic microorganisms. In most of these studies [14,15,16] the synthesized AgNPs demonstrated strong antibacterial activity. For example, in agriculture, AgNPs have been shown to be effective when applied under suitable doses and conditions, promoting plant growth [17] and preventing food spoilage, decay, and discoloration when used as food coatings [18]. In the field of wound healing, various AgNP-based formulations are employed depending on wound type. Their employment has essentially increased due to their strong antibacterial effectiveness against wound-infecting bacteria. In particular, studies have reported AgNPs’ successful application in different wound types, such as burn [19] and scratch models [20] in both *in vivo* and *in vitro* settings. AgNPs have also been applied in oral health, where they help reduce bacterial populations responsible for infections, thereby supporting oral and dental hygiene [21]. The application area of AgNPs further extends to industrial aspects, in particular is water treatment, where AgNPs are used for dye degradation, pathogen removal, and overall water quality improvement [22]. To summarize, AgNPs continue to attract significant attention due to their broad-spectrum antibacterial potential and diverse applicability across both scientific and industrial areas.

Toxicity of AgNPs is considered a major drawback although they have many desirable characteristics. Synthesis method and the physicochemical properties are the main contributors of AgNPs toxicity potential. Physical, chemical, and green synthesis are the three main and most studied synthesis methods to produce AgNPs. Physical synthesis relies on top–down processes to produce NPs without the use of chemical reducing agents. Chemical synthesis utilizes chemical reducing and stabilizing agents that may lead to adverse toxic effects. Green synthesis being an environmentally friendly and sustainable approach, utilizes bioactive compounds from plants, bacteria and fungi to mediate reduction and stabilization of resulting NPs [24,25].

In this review, antibacterial applications of AgNPs, their mechanisms of action, synthesis approaches, application area from agriculture to industrial, and current toxicity concerns are evaluated. We also highlight their expanding applications in fields ranging from agriculture and food systems to biomedical and industrial sectors. Finally, we address current toxicity concerns and emerging challenges that must be resolved for safe and sustainable use of AgNP-based technologies.

## 2. Antibacterial Activity of Silver Nanoparticles

The antibacterial potential of silver ions has been discussed in the literature with a long history [26]. Most recently, silver ions became one of the main research topics in antibacterial research, leading to their inclusion in many studies [6]. Considering the growing potential of drug-resistant bacteria as a global health threat, the requirement for alternative resistance breaker agents becomes extremely important [27]. In this context, AgNPs are recently highlighted with their strong antibacterial activity, biofilm inhibition and effectively acting as antibiotic adjuvants [28,29]. This is why AgNPs have been featured as a promising alternative to conventional antibiotics, considering the growing threat of antibiotic resistance [30,31].

### 2.1. Antibacterial Mechanisms of Silver Nanoparticles

**Figure 2:**
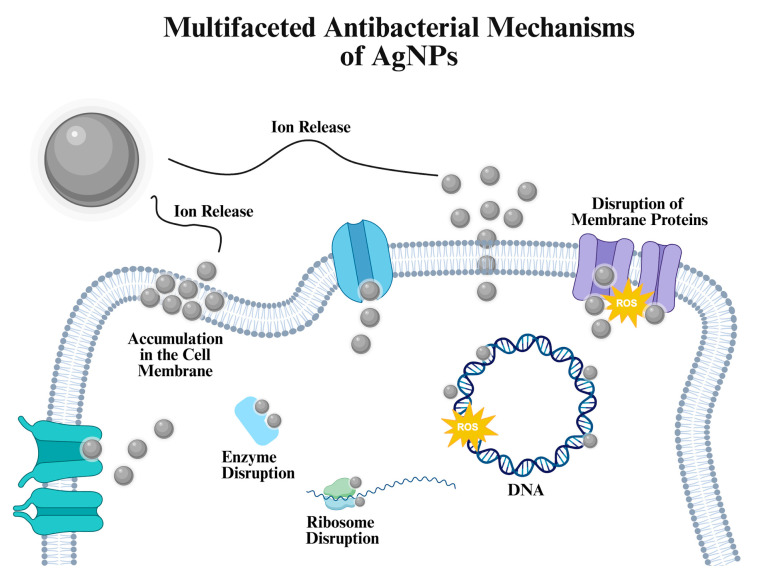
Antibacterial Mechanism of AgNPs. AgNPs exert antibacterial effects through ion release, membrane accumulation, and intracellular disruption. The diagram depicts the release of Ag^+^ ions (small grey spheres) and their subsequent inhibition of membrane proteins and cytosolic enzymes. Yellow starburst icons represent ROS-mediated oxidative stress on DNA and proteins, while directional lines illustrate the Ag^+^ ion penetration pathways [32].

AgNPs exhibit antibacterial effects through several mechanisms that disrupt key cellular processes (Figure 2). These mechanisms may act alone or together, and are outlined in the following subsections.

#### 2.1.1. Mechanisms of Antibacterial Action

Although AgNPs are often discussed as a single antibacterial entity, their biological activity originates from two closely related but mechanistically distinct contributors: (i) the AgNP-mediated effects and (ii) silver ions released from the AgNP surface. AgNP-specific antibacterial effects are primarily associated with direct physical interactions with the bacterial cell envelope, including membrane adsorption, surface-mediated disruption, and NP internalization. In contrast, Ag^+^ ions predominantly demonstrate their antibacterial activity through intracellular interactions with essential biomolecules such as proteins, DNA, and enzymatic systems, leading to impaired metabolic function and cellular homeostasis. The relative contribution of these two components depends on physicochemical properties of the AgNPs, including size, surface charge, coating, and environmental conditions.

*Disruption of the bacterial cell membrane*

The tunable surface charge of AgNPs, which plays a central role in NP-mediated membrane interactions, enables their interaction with negatively charged bacterial membranes, facilitating their antibacterial activity. As the interaction is established, AgNPs are incorporated into the membrane, increasing the permeability. Structural and morphological disruption of the membrane causing loss of functional integrity, resulting in cell death.

Structural differences between Gram-positive and Gram-negative bacteria, including a thinner peptidoglycan layer and the presence of outer membrane porins in Gram-negative strains, can influence their susceptibility to NPs. For instance, mutations affecting the major outer membrane porins in Gram-negative bacterial strains have been shown to increase resistance to AgNPs and silver ions by several-fold, suggesting that porins play an important role in NP uptake [33]. In this context, Radzig *et al.* reported that *Escherichia coli* strains lacking OmpF or OmpC porins were 4 to 8 times more resistant to AgNPs of 8.3 nm compared to wild-type cells, further supporting the role of porins in NP penetration [34].

Wang *et al.* [35] investigated the effect of DAMP-coupled AgNPs’ membrane distribution activity against *Salmonella enterica* ATCC 13311. The small-sized spherical AgNPs, produced with thiolate ligands, effectively exhibited antibacterial activity as novel bacteriostatic compounds. The AgNPs were particularly effective against planktonic *S. enterica* through disrupting membrane integrity, increasing membrane permeability and causing irreversible membrane damage [35].

Researchers investigated molecular interaction between the bacterial cell membrane and AgNPs. It was found that administration of AgNPs influenced the permeability of the *S. enterica* inner cell membrane in a dose-dependent manner, as determined by assays detecting ion leakage and cytoplasmic component release. At lower concentrations of 3.2 and 6.4 μg/mL, AgNPs caused minimal changes, while higher concentrations of 12.8 and 25.6 μg/mL resulting in significantly increased inner membrane permeability and leading to membrane depolarization, intracellular Ca^2+^ accumulation, and eventually bacterial cell death. In contrast, the outer membrane permeability remained largely unchanged, as confirmed by experimental measurements showing no notable change in outer membrane ion uptake [36].

*Release of silver ions (Ag^+^)*

Ag^+^ ions released from AgNPs represent an ion-driven antibacterial pathway distinct from NP-mediated mechanisms. AgNPs can interact with essential molecules such as DNA, lipids, and proteins inside the cell, which affect the systems that are crucial for cell survival. For example, AgNP-induced alterations to ribosomal components can disrupt protein synthesis and lead to irreversible cellular damage. Ag^+^ ions can also damage the structural integrity of cellular proteins by disrupting the disulfide bonds, leading to complete loss of their function. As proteins become misfolded or inactive key processes such as metabolism, signaling, and cellular repair are severely impaired, which reduces the cell survival rate [9].

Sorinolu *et al.* investigated how silver ions released from light-activated AgNPs inhibited the proliferation of antibiotic-resistant bacteria. In the study, they showed that the interaction between AgNPs and photosensitizer protoporphyrin IX (PpIX) may increase the rate of Ag^+^ ion release, which in turn enhances the antibacterial activity. In addition, they investigated the effect of polyethyleneimine (PEI) coating on the bactericidal activity to investigate the effect of positive charge on the AgNP surface. Three different AgNP types, PpIX-AgNPs, AgNPs, and PEI-PpIX-AgNPs were tested against methicillin-resistant *Staphylococcus aureus* (MRSA) and wild-type multidrug-resistant (MDR) *E. coli*. PpIX-AgNPs showed the highest antibacterial activity, achieving more than 7 log reductions in both MDR *E. coli* and MRSA counts. The significance of AgNPs in terms of bacterial log inactivations were PpIX-AgNPs > PEI-PpIX-AgNPs > AgNPs. This ranking was consistent with the measured concentrations of released Ag^+^ ions, confirming the importance of ion release for antibacterial efficiency [27].

*Induction of oxidative stress*

AgNPs trigger oxidative stress through combined effects of NP contact with cellular structures and the release of Ag^+^ ions, both of which contribute to ROS generation. Upon interacting with the bacterial membrane or penetrating into the cell, AgNPs facilitate redox reactions that produce superoxide anions and hydroxyl radicals [37,38]. Nonetheless, excessive ROS generation is a major factor contributing to the toxic effects of AgNPs. While ROS are normally maintained at controlled levels, their overproduction damages DNA, proteins and lipids, which directly contribute to bactericidal potential of AgNPs [39].

Das *et al.* [40] investigated the contribution of ROS to the antibacterial mechanism of AgNPs. Using *Staphylococcus aureus* and *E. coli*, they investigated how ROS generated by AgNPs influenced antibacterial activity. They have emphasized that high ROS levels from AgNP caused significant membrane damage and disruption of the electron transport chain [40]. Ali *et al.* [41] examined the antibacterial activity of AgNPs synthesized using aqueous extracts of *Origanum vulgare* (O-AgNPs), *Salvia moorcroftiana* (S-AgNPs), ceftriaxone (Cfx-AgNPs) to investigate ROS-based effects. The synthesized AgNPs were tested against *Klebsiella pneumoniae* and *Pseudomonas aeruginosa*, where the *K. pneumoniae* was found to be the most susceptible strain during the treatment. For the Cfx-AgNPs, *P. aeruginosa* exhibited the highest response, with a Channel S1 (ChS1) count of 56.865. These results demonstrated that Cfx-AgNPs induced the highest levels of ROS, leading to notable DNA, protein degradation and cellular damage [41].

*Interaction with DNA and ribosomes*

AgNP-mediated interference with DNA and ribosomal function occurs through a combination of direct NP-cell interactions and the activity of Ag^+^ ions released from their surface. Together, these contributions enable AgNPs and Ag^+^ ions to bind intracellular biomolecules, disrupt structural integrity, and impair normal cellular processes. AgNPs strongly interact with both DNA and proteins, binding to nucleobases such as cytosine as well as to *N*-terminal amines and other metal-affinity sites within the cell. In parallel, AgNPs and released Ag^+^ ions form strong coordination bonds with sulfur-containing residues, most notably cysteine and methionine, in proteins, which destabilizes protein structure and function and can amplify downstream cellular damage. These alterations negatively affect cellular mechanisms such as respiration and cell division that can lead to cell death. As a result, the cell suffers genotoxicity, mutation, DNA strand breaks and oxidative DNA base damage. AgNPs disrupt cellular homeostasis of *E. coli* through DNA damage and induced mutations in genes that are responsible for DNA repair. AgNP–protein interactions also disrupt functional capability of proteins and inactivating enzyme activity. The disruption of the 3D structure of proteins inactivates the binding sites of proteins. As a result, AgNPs can effectively restrict cellular activity by binding to and deactivating proteins [42].

A comprehensive study investigated the interaction between DNA and green-synthesized AgNPs using *Helianthemum lippii* extracts [43]. The results showed that negative free binding energy was associated with the increased DNA binding, causing spontaneous interactions. The electrostatic interaction with the ligands is an important factor that contributes to the DNA degradation. These mechanisms highlight the therapeutic potential of AgNPs against infectious diseases and conditions involving uncontrolled cell growth, including cancer.

#### 2.1.2. Synergistic Antibacterial Activity with Antibiotics

AgNPs stand out with their ability to inhibit proliferation of multidrug-resistant (MDR) strains [44]. Apart from their sole administration, AgNPs can be combined with antibiotics to obtain enhanced bactericidal effects, where they can improve the efficiency of antibiotics [45]. The potential of antibiotics is affected by proper dosing. Their insufficient or excessive administration contributes to MDR. In this manner, AgNP enhances antibiotic effectiveness by allowing utilization of lower and safer therapeutic doses against MDR bacteria [46].

A recent study highlighted the synergistic antibacterial effect of AgNPs with antibiotics. The study was conducted using several different bacterial species. When AgNPs (100 μg per disc) were applied in combination with colistin (10 μg per disc), the inhibition zones were measured as 20.27 ± 0.58 mm for *Acinetobacter baumannii*, 34.94 ± 0.39 mm for *E. coli*, 27.18 ± 0.42 mm for *K. pneumoniae* and 19.16 ± 0.53 mm for *P. aeruginosa*. However, treatment with colistin alone produced inhibition zones of 13.89 ± 0.52 mm, 25.18 ± 0.26 mm, 20.52 ± 0.48 mm, and 16.43 ± 0.18 mm for *A. baumannii*, *E. coli*, *K. pneumoniae* and *P. aeruginosa*, respectively. When working together, colistin reduces the permeability of the cell membrane. AgNPs then easily pass through the cell membrane and cause the cell membrane to break down. AgNPs that reach the inside of the cell cause ROS production by damaging the cell’s DNA, facilitated by colistin. This ultimately leads to cell death. These findings demonstrate the enhanced antibacterial activity of combined colistin and AgNPs [47]. Shahverdi *et al.* [48] tested 14 different antibiotics to assess their effectiveness against *E. coli* and *S. aureus* in combination with polydisperse AgNPs. The antibacterial tests revealed that most notable increases in antibacterial activity were observed for vancomycin, amoxicillin, and penicillin G. It was found that penicillin G alone had no inhibitory effect against *S. aureus*, whereas its combination with AgNPs produced a 12 mm zone of inhibition. Similarly, vancomycin alone had no inhibitory effect against *S. aureus*, but when combined with AgNPs, the zone of inhibition was 13 mm. The most significant increase was observed for amoxicillin, where the inhibition zone expanded from 7.5 mm when used alone to 14.0 mm when combined with AgNPs. This article does not explain exactly how the antibacterial mechanism works. It also adds that this is worth researching [48]. Together, these results show that the synergistic activity of AgNPs with antibiotics is not limited to a single drug or organism. While the first study demonstrated improved inhibition across multiple Gram-negative pathogens using colistin, the second showed that AgNPs can enhance the effectiveness of several different antibiotics, even restoring activity to drugs that are normally ineffective against resistant strains

Similarly, Lee and colleagues investigated the combined antibacterial effects of various antibiotics (aminoglycoside, streptomycin, gentamicin, tobramycin, kanamycin) combined with AgNPs, demonstrating their combined efficacy against pathogenic *E. coli.* Comparison of minimum inhibitory concentration (MIC) and minimum bactericidal concentration (MBC) values revealed that AgNP-antibiotic combinations increased antibacterial effects. Furthermore, the AgNP–tobramycin combination achieved the highest biofilm inhibition rate of 19.87% among the antibiotics tested. In this study, antibiotics with synergistic effects with AgNPs cause the death of microorganisms by weakening the cell membrane, disrupting DNA and protein synthesis, and silencing resistance-related genes. The study concluded that the combination of aminoglycosides and AgNPs increases antibacterial potential [49]. Smekalova *et al.* investigated the synergistic effect of AgNPs and antibiotics, penicillin G, colistin, amoxycillin and gentamicin, against several animal pathogens including *S. enterica*, *E. coli*, *S. aureus*, *Streptococcus uberis*, *Pasteurella multocida*, and *Actinobacillus pleuropneumoniae* [50]. Researchers identified 7 synergistic, 17 additive and 16 indifferent effects in the tested AgNP–antibiotic combinations, with no antagonistic effects observed. Among these combinations, AgNP–gentamicin had the majority of synergistic effects, while the strongest synergistic effect observed against *A. pleuropneumoniae* by 6.3 µg/mL AgNP−8 nm and 2 µg/mL penicillin G combination, with a fractional inhibitory concentration (FIC) index of 0.3. Additionally, the combined AgNP treatments restored susceptibility in *A. pleuropneumoniae* and *P. multocida*, which were originally resistant to gentamicin, amoxycillin, and colistin. From another perspective, Adil *et al.* [51] synthesized antibiotic-supplemented AgNPs in a 1:1 molar ratio, utilizing cephalosporin class antibiotics including cefepime, ceftazidime, ceftriaxone and cefotaxime. Results highlighted enhanced antibacterial activity against K. *pneumoniae*, *P. aeruginosa*, *S. aureus*, and *Enterococcus faecalis* by AgNP–antibiotic conjugates, in comparison to antibiotics or AgNPs alone, with the exception of the cefepime–AgNPs. The authors attributed these enhanced antibacterial effects to the antibiotic coating that surrounds AgNPs, which increases local antibiotic concentration at the target side [51]. Collectively, these studies further expand evidence for AgNP–antibiotic synergy across a wide range of drug classes and bacterial targets. While aminoglycoside combinations were shown to enhance antibacterial and antibiofilm activity primarily against *E. coli*, broader studies demonstrated synergistic or additive effects across multiple clinically relevant pathogens, including *S. enterica*, *S. aureus*, and *P. multocida*. Notably, no antagonism was reported, and in several cases susceptibility was restored in strains previously resistant to conventional antibiotics. In addition, approaches using direct antibiotics–AgNP conjugates have shown that surface-bound antibiotics can improve local drug delivery and enhance bacterial killing compared with either agent alone. Together, these findings support AgNP–antibiotic combinations as a promising strategy for improving antimicrobial efficacy and overcoming resistance.

### 2.2. Effect of Physicochemical Properties of AgNPs in Antibacterial Activity

AgNPs exhibit outstanding physicochemical properties, such as size, high surface area-to-volume ratio and notable electrical, thermal, and optical conductivity. They also display considerable diversity depending on their synthesis methods, experimental conditions and structural characteristics [23]. Particle size influences the physicochemical characteristics of AgNPs and their biological interaction capability. Furthermore, it can play an important role in their toxicity potential, since larger particles are known with lowered cytotoxicity potential as a result of their smaller relative surface area. Even though small-sized AgNPs exhibit higher toxicity potential with potent reactivity, they also show the most efficient antibacterial activity and biofilm inhibition, as they can efficiently penetrate cells. The bactericidal activity of AgNPs is highly affected by their morphology, including their interaction with cellular structures. Depending on the particle’s surface charge, the antibacterial efficiency is also influenced since their interaction with bacterial cell membranes is mediated by electrostatic interactions. Moreover, tunable surface chemistry of AgNPs can be manipulated to enhance the antibacterial activity, improving stability and antibacterial potency [6].

*Effect of Size on Antibacterial Activity*

The relationship between the particle size and the antibacterial potential of AgNPs are thoroughly discussed [52]. Depending on the size, AgNPs can penetrate the bacterial cells efficiently, thus facilitating their intracellular antibacterial mechanisms.

Wu *et al.* [53] synthesized AgNPs with different sizes (2, 12 and 32 nm), testing against *E. coli* and *S. aureus*. Results highlighted that small-sized AgNPs demonstrated higher antibacterial activity with lower MIC values and larger inhibition zones [53]. Similarly, Ershov *et al.* [54] demonstrated the enhanced biocidal activity of small-sized AgNPs. The MIC and IC_50_ values were approximately 1.3 × 10^−4^ M and 6.5 × 10^−6^ M for 22.7 nm particles, and 0.6 × 10^−4^ M and 3.0 × 10^−6^ M for 10.8 nm particles, respectively. Half-maximal inhibitory concentration (IC_50_) values were similar and related to the particle size. Results suggested that smaller particles equipped with a greater reactive surface area, enhancing antibacterial activity [54].

*Effect of Shape on Antibacterial Activity*

In addition to size, the morphology of AgNPs also has a significant impact on their antibacterial potential [6]. NPs can adopt various shapes, including spherical, rod, triangular, cubic and star-like forms, depending on the synthesis method [55]. The interaction between the bacterial cell membrane and the NP is influenced by the shape, which can enhance or reduce overall antibacterial effectiveness [6]. Shape also affects the release of ions from NPs, further contributing to antibacterial activity [56]. Among these shapes, spherical NPs are particularly advantageous due to their higher surface area-to-volume ratio compared to other types of NPs [55]. Overall, the morphology of AgNPs determines their mode of action on bacterial cells and positions them as versatile and widely used antibacterial agents [6].

In a study on this subject, antibacterial activity of AgNPs of different shapes, spherical (AgNSs), triangular plate (AgNTs) and disc (AgNDs) were investigated against *E. coli*, *S. aureus*, and *P. aeruginosa*. The AgNPs showing the largest zone of inhibition against *E. coli* was AgNSs with 4.8 mm, while AgNDs and AgNTs showed weaker effects. Based on their antibacterial effects, the ranking was as follows: AgNSs demonstrated the highest activity, AgNDs showed moderate activity and AgNTs had the weakest effect. [57]. Similarly, Helmlinger *et al.* [58] investigated the effect of NP shape on antibacterial activity. They synthesized AgNPs with uniform shape and size, including nanospheres (40–70 nm and 120–180 nm in diameter), nanoplatelets (20–60 nm), nanocubes (140–180 nm) and nanorods (80–120 nm in diameter). The antibacterial activity was further evaluated against *S. aureus* via MBC measurements. Among various morphologies, nanoplatelets demonstrated the strongest antibacterial activity. These results indicate that the antibacterial potential of AgNPs is greatly affected by their morphology, including their dissolution rate and surface Ag^+^ ions [58]. From another perspective, Parit *et al.* [59] tested the antibacterial potential of green-synthesized AgNPs with various morphologies, including spherical, hexagonal and triangular. These AgNPs, which come in a wide variety of shapes, have shown strong antibacterial effects against the bacterial strains used in the study [59]. Hong *et al.* [60] examined how the shapes of AgNPs affect antibacterial activity. They used specifically nanocubes, nanospheres, and nanowires AgNPs. According to this study, the first factor to consider is the effective surface area. Accordingly, nanowires establish the weakest contact with cells due to their one-dimensional nature and low specific surface area. This results in low antibacterial activity. In contrast, nanocubes showed higher efficiency. Experiments revealed that at a concentration of 12.5 µg/mL, nanocubes delayed bacterial growth for up to 7 h, while no such effect was observed with nanowires or nanospheres. This study demonstrates that the shapes of AgNPs play a significant role in their antibacterial effect [60]. Acharya *et al.* [61] examined the antibacterial effects of spherical, rod-shaped, triangular, and hexagonal AgNPs. The most fundamental result of this study shows that spherical AgNPs have the highest antibacterial activity and the fastest killing rate compared to other shapes. In the experiments, spherical AgNPs were tested on *K. pneumoniae* bacteria. According to the results obtained, a mortality rate of 76% or higher was observed within 240 min. This rate remained at 23% for rod-shaped AgNPs, 15% for hexagonal AgNPs, and 15% for triangular AgNPs. The article also states that this high effect stems from the release rate of Ag ions in spherical AgNPs. The highest release rate is 34 µg/mL in spherical shapes. This also shows us the importance of Ag ion release along with shape [61]. These studies highlight that AgNP antibacterial activity varies widely with particle morphology. Spherical, disc-shaped, nanocube, and platelet-like AgNPs consistently demonstrate stronger inhibition than rods, wires, or triangular structures, likely due to greater surface contact and higher Ag^+^ ion release. Green-synthesized AgNPs with varied geometries also exhibit notable activity, reinforcing that surface area and exposed crystal facets strongly influence bacterial killing. Overall, morphology serves as a key determinant in optimizing AgNP antibacterial performance.

*Effect of Zeta Potential and Colloidal Stability on Antibacterial Activity*

The colloidal stability and antibacterial activity of AgNPs are related to the surface charge and zeta potential of the particles. Research indicates that a zeta potential value greater than +30 mV or less than −30 mV increases particle stability. This stability prevents agglomeration. Accordingly, AgNPs with zeta potentials within this range exhibit higher antibacterial effects. The effect of zeta potential on antibacterial activity has been investigated in the literature [62].

A study by Salazar-Bryam *et al.* [63] describes the effect of pH changes during synthesis on AgNPs. According to this study, the stability of AgNPs was analyzed using zeta potential. An increase in pH from 5 to 9 during synthesis resulted in an increase in zeta potential (from −29.86 mV to −40.33 mV). Furthermore, more stable AgNPs were obtained at higher pH values. As a result of this stability, the more stable AgNPs exhibit a strong synergistic antibacterial effect with rhamnolipids [63]. Rónavári *et al.* [64] examined the behavior of AgNPs coated with polyvinylpyrrolidone (PVP) under various conditions in their studies. One of the important findings of this article is that the PVP coating provided steric stabilization to the AgNPs. As a result of this stabilization, they were found to be more resistant to biological agglomeration compared to electrostatic stabilization. In addition, the chemical stability of the particles was also examined. The formation of silver chloride (AgCl) precipitates in the presence of chloride ions (Cl^−^) was investigated. Although colloidal protection is provided, complete chemical protection has not been achieved [64]. In another study conducted by MacCuspie *et al.*, they investigated how the physicochemical properties of AgNPs affect colloidal stability under relevant conditions [65]. They examined the stabilization provided to AgNPs by coating materials such as citrate, bovine serum albumin, and starch, which are surface coating agents for AgNPs. Although citrate coatings provide electrostatic stabilization, they become ineffective in the face of high ionic strength. The particles lose their effect by being grounded. Steric stabilization is provided in bovine serum albumin coating. With this coating, particles can remain individual and active for up to 64 h in various environments. In addition, the increased surface area compared to bulk silver, combined with natural solubility, has affected antimicrobial activity. This increase in surface area leads to greater ROS production and DNA damage in bacteria. These coatings give AgNPs powerful antibacterial properties.

*Effect of Surface Chemistry and Capping Agents on Antibacterial Activity*

Silveria *et al.* [66] synthesized AgNPs from the *Ilex paraguariensis* plant. By examining the physicochemical properties of these synthesized AgNPs, they focused on their antibacterial effect [66]. One of the results obtained in this study was obtained through FT-IR analysis. According to these results, functional groups such as polyphenols in the plant extract reduced the Ag^+^. This reduction resulted in the formation of a protective coating around these particles. Thanks to this coating, the particles became more stable. The antibacterial activity of these stable AgNPs was tested on *E. coli* and *S. aureus* bacterial strains. The results showed that inhibition was achieved even at high concentrations. Sinclair *et al.* focused on the antibacterial effect of the surface chemistry of AgNPs in their study [67]. Various coating materials were used in this study. The first of these materials is positively charged branched polyethyleneimine (BPEI). AgNPs coated with BPEI showed the highest effect on MS2 bacteriophages. This is due to the electrostatic attraction caused by the negative surface of these bacteriophages and the positive charge on the surface of AgNPs. AgNPs were also coated with substances that can create a strong negative surface, such as citrate and mercaptoacetic acid (MAA). As a result, there was more interaction with positively charged microorganisms. Since these coatings will come into direct contact with the target organism, there is an increase in antibacterial and biological activity.

*Effect of Surface Charge and Functionalization on Antibacterial Activity*

Surface charge is essential for NPs to attach to and interact with bacterial cell membranes. It plays a critical role in determining how NPs engage with bacterial membranes and proteins. Surface charge is typically measured using zeta potential, and whether the charge is positive or negative significantly influences AgNPs’ interactions [68].

Gulami *et al.* [69] were able to synthesize a positively charged silver nanocomplex for chlorhexidine (CHX) using ionic liquids in their study. The nanocomplex produced in this study can deliver the antibacterial agent in a synergistic manner. This synthesized positively charged silver nanocomplex (AgNPs+) and CHX-loaded positively charged AgNPs (CHX@AgNPs) were synthesized and characterized by various means. They found the zeta potentials of AgNPs+ and CHXAgNPs+ to be +18 mV and +56 mV, respectively. To summarize, they observed that the MBC and MIC values were lower than those of AgNPs+ and CHX alone. With this result, they concluded that it could be a potential root canal disinfectant. However, they stated that more ex vivo studies are needed to fully incorporate this antibacterial agent into our lives [69]. In another study, five types of AgNP colloids with identical sizes and shapes but different surface ligands were synthesized, resulting in zeta potentials ranging from −47.6 mV to +68.5 mV. Later, AgNPs were tested against various Gram-positive and Gram-negative bacterial species, including *S. aureus*, *E. faecalis*, *E. coli*, *P. aeruginosa*, *A. baumannii*, and *K. pneumoniae*. Results revealed that AgNPs with intermediate charges (−21.5 mV and +14.9 mV) exhibited stronger antibacterial activity than those with extreme positive or negative charges. Additionally, Gram-positive bacteria were more sensitive to higher positively charged AgNPs, whereas Gram-negative bacteria were more sensitive to higher negatively charged AgNPs [70].

Surface functionalization involves attaching organic or inorganic biomolecules to AgNP surfaces through covalent or non-covalent interactions. Such functionalized NPs, characterized by improved stability and performance, have been widely employed in antibacterial applications [71].

Owing to their high surface area, AgNPs provide a major advantage for tunable surface functionalization and can interact with diverse organic molecules and biomolecules. As an example, AgNPs can form strong Ag-S linkages with amino acids, peptides, and proteins, while also engaging with functional groups containing nitrogen and oxygen. Moreover, AgNPs can bind into the organic compounds containing functional groups such as phenol, carbonyl and hydroxyl, increasing their antibacterial potential [72].

As an example, Khan *et al.* [73] compared the antibacterial efficiency of AgNPs with and without functionalization using polyethylene glycol (PEG). Three different PEG molecular weights (750, 2000, and 5000) were used for surface coating, referred to as P750-, P2000-, and P5000-AgNP, respectively. These NPs were tested for bactericidal activity against *S. aureus*. Findings revealed PEG-functionalized AgNPs’ stronger antibacterial activity compared to non-functionalized AgNPs. P5000-AgNPs achieved an inhibition zone of 29 mm, compared to 22 mm for bare NPs. It was also reported that smaller-sized AgNPs combined with higher molecular weight PEGs were more effective in inhibiting the proliferation of *S. aureus* [73]. In another study, Veena *et al.* [74] functionalized AgNPs with 4-amino-3,5-dimercapto-1,2,4-triazole (DMT) to enhance intracellular uptake and biocompatibility. The functionalized AgNPs showed strong antibacterial activity against both Gram positive and Gram-negative bacteria strains. In addition, researchers also investigated the anticancer potential of DMT-AgNPs, revealing the cell cycle arrest and apoptosis in MCF-7 cancer cells in a dose-dependent manner (IC_50_ value of 18.46% *v*/*v*). Overall, DMT functionalization enhanced the antibacterial potential of AgNPs, along with their anticancer activity [74]. Shumi *et al.* [75] synthesized AgNPs functionalized with the amino acid histidine (AgNP-His) and phenylalanine (AgNP-Phen) using *Lippia abyssinica* leaf extracts through a green synthesis approach. Both types of AgNPs exhibited antibacterial activity against *E. coli* and *S. aureus*, with inhibition zones of 8.67 ± 1.25 mm and 11.00 ± 0.82 mm for AgNP-His, and 8.33 ± 0.47 mm and 8.33 ± 1.25 mm for AgNP-Phen at a concentration of 62.5 μg/mL, respectively. Overall, the study indicated that amino acid functionalization may represent a promising strategy for developing novel antibacterial agents [75].

### 2.3. Fundamental In Vitro Studies

Numerous studies in the literature have investigated the *in vitro* antibacterial effects of AgNPs. These studies generally evaluate the effects of AgNPs on bacterial species using various experimental parameters. Examples of these parameters include MIC and MBC values. These values provide clear evidence of whether AgNPs act as strong antibacterial agents.

Yusuf-Salihu *et al.* [76] conducted a comprehensive study investigating the antibacterial activity of AgNPs synthesized from empty fruit bunches (EFB) waste. The researchers applied a green synthesis approach, converting EFB waste into high-value AgNPs, combining waste valorization with NP production Their antibacterial, antioxidant, antidiabetic, anticoagulant, and thrombolytic activities were investigated, marking the first reported successful biosynthesis of AgNPs from EFB. The NP formed ranged in diameter from 12.78 to 19.10 nm. These NPs exhibited notable dose-dependent activity against several bacteria species: *E. coli*, *Klebsiella oxytoca*, *S. aureus*, *Proteus mirabilis*, and *P. aeruginosa*, with inhibition rates between 40% and 80%. They also achieved 100% inhibition against *Aspergillus niger*, *Aspergillus fumigatus*, *and Aspergillus flavus*. The study also showed that the NPs induced oxidative stress through ROS generation. In later stages of the study, antioxidant capacity of AgNPs was also examined. Overall, high-value EFB-based AgNPs were successfully synthesized, offering both waste valorization and effective antibacterial activity [76]. Koçer *et al.* [77] synthesized AgNPs using macroalgae and evaluated their antifungal and antibacterial activities. The synthesis was carried out using green methods with extracts from *Ulva lactuca* and *Halopteris scoparia*. In terms of antibacterial activity, inhibition zones of 10–15 mm in diameter were observed. The antifungal activity of these macroalgae-derived AgNPs was also examined and found to be comparable to their antibacterial activity. With this study, it has been seen that macroalgae-based green synthesized. The findings demonstrate that macroalgae-based green-synthesized AgNPs exhibit strong antibacterial activity and offer an environmentally friendly alternative to chemically and physically produced NPs [77].

Kavin *et al.* [78] synthesized AgNPs using *Coffea arabica* husks and investigated their enhanced antibacterial and anticancer activities. The synthesized NPs exhibited spherical morphology with an average size of 147 nm were obtained. When tested against *P. aeruginosa*, *Bacillus subtilis*, *E. coli*, and *S. aureus*, the inhibition zones measured 8.2, 5.3, 4.0, and 3.8 mm, respectively. For Gram-positive *S. aureus* and *B. subtilis*, the MBC and MIC values were 1.5 and 0.75 mg/mL, respectively. For Gram-negative *P. aeruginosa*, the MBC was 0.75 mg/mL and the MIC was 0.37 mg/mL. Based on this result, AgNPs synthesized from the *Coffea arabica* plant were considered important agents for antibacterial activity. The study also demonstrated that these AgNPs exhibited cytotoxic effects against MCF-7 cancer cells [78]. Similarly, Yazdi *et al.* [79] conducted a comprehensive study using AgNPs synthesized from *Lentinus tigrinus* mushrooms and examined the synergistic antibacterial effects in combination with the acetone extract of *L. tigrinus*. AgNPs were 5 to 25 nm in size and had spherical morphology. Bare NPs were only able to inhibit proliferation of *P. aeruginosa* below concentration of 1 mg/mL. When combined with acetone extracts, AgNP–extract combination at a concentration of 0.5 mg/mL had increased inhibition of 380% against *S. aureus*, 306% against *E. coli* and 900% against *Bacillus cereus* in compared to bare AgNPs [79]. Das *et al.* [80] investigated the bactericidal potential of AgNPs green-synthesized from *Trema orientalis* extracts. NPs had a size range between 14.04 and 34.38 nm and were spherical in shape. AgNPs had a MIC value of 55.31 µg/mL against *S. aureus*, along with Inhibition zones of 9, 10, 13 and 14 mm at 25, 50, 75, and 100 µg/mL [80].

Overall, these *in vitro* studies provide the necessary scientific understanding of how AgNPs act against bacteria under controlled laboratory conditions. Through determination of MIC and MBC values and showing how different bacteria respond in AgNP treatment, these results contribute to the explanation of how in what degree AgNPs can be effective as antibacterial agents. Based on this foundation, the next section focuses on how AgNPs are applied in wide-ranging antibacterial fields.

## 3. Antibacterial Applications of Silver Nanoparticles

AgNPs have been primarily applied in antibacterial treatments, agriculture, wound healing, dentistry, and wastewater treatment, arising from their strong antibacterial action. Continued research on AgNPs is essential to advance these fields, with a strong emphasis on developing NPs that can be efficiently integrated into commercial and practical applications (Table 1).

### 3.1. Agricultural Applications of AgNPs

AgNPs are recognized as sustainable alternatives to conventional pesticides due to their strong antimicrobial activity, surface reactivity and multiple modes of action. Yet, conventional chemical synthesis relies on chemicals with dangerous potential to the environment, making AgNPs less suitable for agricultural applications. Green synthesis routes reveal high potential to reduce the environmental impact of chemically synthesized AgNPs. This enables suitable implementation of AgNPs that target plant pathogens. Food packaging is another application where AgNPs’ immense antibacterial characteristics can be exploited through their integration into agriculture and food systems. In this way, AgNPs ability to support more resilient and sustainable production and reduce pesticide dependence presents a promising strategy [105].

AgNP and sweet potato anthocyanin (PSPA) loaded Chitosan/polyvinyl alcohol films were developed by Wu *et al.* [106] to investigate their ability on strawberry preservation. Incorporation of AgNPs with PSPA improved the functional, barrier and physical properties of the film. Strawberries coated with PVA/CS-AgNPs-PSPA10 film remained visibly intact for up to 13 days of storage, which is attributed to AgNPs’ antibacterial activity where they effectively inhibited bacterial growth. In contrast, uncoated strawberries showed visible spoilage after 2 days, whereas those coated with only PVA/CS or wrapped in regular stretch film exhibited spoilage after 5 days. As a result, the composite film effectively preserved fruit freshness, reduced bacterial growth and delayed spoilage [106]. Rutkowski *et al.* [107] investigated the antibacterial activity of AgNPs with sodium alginate hydrogels on red cabbage seedlings, revealing their effect on plant performance. Hydrogels containing AgNPs were tested against several bacterial strains and their effect on seedling growth were determined. AgNP concentrations of 20 and 60 mg/L showed no phytotoxic effects or negative impact on seedling development, while exhibiting potent antibacterial activity. Moreover, while carotenoid levels in the control group increased by 0.107 ± 0.009 mg/g FW, the AgNP-treated group showed a significantly higher increase of 0.134 ± 0.004 mg/g FW. As a result, AgNP–alginate composites exhibited strong antibacterial activity without reducing the plant quality [107]. Together, these findings show that AgNP-based materials can protect crops and fresh produce by inhibiting microbial spoilage, extending shelf life, and improving plant performance without causing harmful effects

Exposure to AgNPs may lead to triggering of physiological responses linked with improved tolerance to drought and enhanced photosynthesis. In this manner Ding *et al.* [108] utilized AgNPs to increase drought resistance in wheat plants. Results showed ROS generated by AgNPs induced mild stress in the plant, enabling the preparation of its defense system to effectively respond following stress conditions. Low concentration of AgNPs (1 mg/L) had no adverse effects while phytotoxic responses were observed at higher levels of administration (5 and 10 mg/L). Treatment also led to a measurable increase in yield-related traits such as grain weight and number by 12%. The study also showed that AgNPs did not accumulate in plant tissues, further supporting their potential safe use [108]. AgNP–spirulina extract formulations were developed by Elbanna *et al.* [109] as an eco-friendly nanofertilizer. Formulations containing different concentrations of AgNPs, 50, 100 and 150 mg/L positively affected height, growth and number of leaves of French basil. However, the highest concentration of AgNPs led to accumulation of malondialdehyde, an indicator of oxidative stress. The synergistic application reduced the ROS-related damage by activating the plant’s antioxidant defense system, thus antioxidant enzyme activation suppressed the excessive ROS levels. The highest yield and positive effect were observed at the concentration where spirulina extract and AgNPs were synergistically present, specifically at 100 mg/L AgNP + 1.5 g/L spirulina extract. These findings suggest that synergistic application of AgNPs were more effective than sole application, enhancing basil quality and yield. The mitigated oxidative damage and maintained antibacterial activity highlight the potential of the combination as a nanofertilizer [109]. Aguirre-Noyola *et al.* [110] cultivated strawberries *in vitro* to evaluate the effects of green-synthesized AgNPs on plant development. Green synthesized AgNPs were applied *in vitro* to assess their impact on photosynthetic pigment production and growth parameters, along with the shoot height, leaf number, and total chlorophyll content. The average number of shoots in the control group was determined as 1.50 ± 0.23, while there was a notable change with AgNP treatment in a dose-dependent manner. AgNP treatment with 100 mg/L concentration nearly doubled the number of shoots to 3.08 ± 0.64. However, increased concentrations to 300 mg/L inversely affected the number of shoots, showing a dramatic reduction to an average of 2.00 ± 0.24. Total chlorophyll levels were also evaluated, reaching the highest value at approximately 0.3 mg/g FW at 300 mg/L AgNPs [110]. These findings indicate that AgNPs can promote crop vigor by enhancing stress responses, shoot formation, and pigment production, but that their benefits depend on careful dose control to avoid oxidative damage and growth inhibition.

### 3.2. Biomedical and Healthcare Applications of AgNPs

#### 3.2.1. Wound Healing Applications of AgNPs

One of the key objectives in wound management is to ensure rapid and efficient healing while minimizing bacterial contamination. In parallel, use of AgNPs as alternatives to traditional treatments has notably increased due to their promising antibacterial potential. With their strong antibacterial activity and ability to support tissue regeneration, AgNP-based dressings have become widely utilized in current wound care strategies [111,112]. However, potential cytotoxic effects of AgNPs must be considered, as they have limited their therapeutic applications in some cases [30].

Aldakheel *et al.* [113] developed an AgNP-based hydrogel, aimed to prevent the spread of infection, and accelerate healing by preventing traditional wound dressings, such as poor barrier function and adherence to the wound. AgNPs were synthesized using matcha green tea extract (*Camellia sinensis*) as a green catalyst, further incorporated into chitosan and alginate matrices to produce hydrogels with strong antibacterial properties. Their antimicrobial activity was evaluated through inhibition zone assays against three bacterial and two fungal strains. The AgNP-containing hydrogels exhibited superior water retention, maintained wound moisture and effectively absorbed wound exudate, in comparison to their counterparts containing only chitosan or alginate [113]. Moayedi *et al.* [114] developed nanofibrous burn dressings composed of polylactic acid (PLA), curcumin (Cur), and AgNPs with different formulations (PLA, PLA + Cur, PLA + Cur + 1% AgNP, PLA + Cur + 2% AgNP, and PLA + Cur + 3% AgNP) and evaluated their wound healing efficacy. PLA + Cur + 3% AgNP showed highest curcumin release while PLA+Cur+2% AgNP formulation had the least. The PLA + Cur dressing led to 84.06% destruction of bacteria, while PLA + Cur + 3% AgNP formulation achieved 99.12%. These confirmed the strong antibacterial potential of AgNPs [114]. Sharma *et al.* [115] demonstrated the wound healing potential of green synthesized AgNPs incorporated to 0.5% Carbopol hydrogel. The AgNP–hydrogel was tested in excision wound models, demonstrating efficient wound closure rates. *In vivo* assays on Wistar rats further revealed that hair follicle regrowth and enhanced skin regeneration were greatly increased by the AgNP hydrogel compared to the control group [115]. These findings confirm that AgNP-loaded dressings offer both antibacterial action and accelerated wound closure, supporting their promise for clinical wound management

Bacterial biofilm formation is another major factor that hinders the healing rate of wounds. Gangwar *et al.* [116] conducted a comprehensive study, evaluating the antibacterial and wound healing effects of green synthesized AgNPs. Antibacterial activity was investigated using zone inhibition and MTT assays against *E. coli* and *S. aureus*. Green-synthesized AgNPs were prepared by reacting an optimized and constant concentration of AgNO_3_ (0.2 M) with *Aloe vera* extracts at varying concentrations (125, 250, 375, and 500 mg/mL), where *A. vera* acted as the reducing and stabilizing agent. Zone inhibition results confirmed that the AgNPs were effective against both strains, with highest antibacterial activity in the group containing 0.2 M AgNO_3_ + 500 mg/mL *A. vera*. Wound-healing effects were further evaluated using a scratch assay on mouse fibroblast cells. As a result, AgNP treatment significantly enhanced fibroblast migration and scratch closure, leading to improved wound repair [116]. *In vitro* and *In vivo* wound healing efficiency of AgNPs investigated by Baveloni *et al.* [117] MIC values of 6.74 μg/mL against *S. aureus* and *P. aeruginosa* and 58.5 μg/mL against *E. coli* were indicated through *in vitro* assays. Further *in vivo* studies revealed AgNPs’ efficiency in controlling the microbial growth in wounds infected with *S. aureus* on Wistar rats. These highlighted the potential utilization of AgNPs in treating infections and wound healing studies due to their strong antibacterial potential [117]. Results from *in vitro* scratch assays and *in vivo* infection models suggest that AgNPs can accelerate tissue regeneration while suppressing pathogenic bacteria, positioning them as promising candidates for wound-care therapies.

#### 3.2.2. Dental and Oral Healthcare Applications of AgNPs

AgNPs are highly promising for inhibiting microbial colonization, reducing implant-related infections and limiting multidrug-resistant bacterial growth [118]. Still, dose of administration is essential for safety and efficacy, since excessive amounts can cause cytotoxicity and limit clinical applicability [119].

Téllez Girón *et al.* [120] investigated the anti-caries and anti-adhesive properties of green synthesized AgNPs. The antibacterial activity was evaluated through measurement of acid production and bacterial adhesion of *S. mutans* and *Lactobacillus acidophilus*. The MIC and MBC values for *S. mutans* were 6.68 and 13.17 μg/mL, while the values were 3.34 and 3.34 μg/mL for *L*. *acidophilus*, respectively. In adhesion tests, fluoride varnish applied with AgNPs and exhibited potent inhibition of bacterial adhesion. The combination also reduced bacterial adhesion and acid production associated with the tooth decay. These results highlight that AgNPs effectively demonstrate synergistic antibacterial activity against key cariogenic bacteria as an alternative to conventional dental caries prevention strategies [120]. Kamel *et al.* [121] conducted an *in vivo* study evaluating the antibacterial and regenerative effects of AgNPs on dental pulp. Three experimental groups were designed for the research: group 1 was treated with a resin composite containing 1% (*w*/*w*) AgNPs, group 2 received mineral trioxide aggregate (MTA) and control group was treated with conventional resin. Histomorphometric analysis showed an inflammatory cell area of 43.93 ± 1.87% in the control group, which decreased to 20.12 ± 1.20% in Group 2 and 14.7 ± 1.07% in the AgNP group. AgNP treatment did not induce any necrosis and managed reparative dentin formation in continuous [121]. Synergistic employment of AgNPs with polymethyl methacrylate (PMMA) for dental applications were investigated by Ayuso *et al.* [122] PMMA nanocomposites were incorporated with green-synthesized AgNPs to improve bactericidal potential. PMMA formulation containing 10 mg/mL AgNPs achieved decreased biofilm adhesion of 18.2 ± 2.8% compared to sole PMMA formulation having 91.6 ± 4.7%. For Opti-Cryl PMMA, biofilm adhesion decreased from 49.5 ± 3.3% to 16.7 ± 2.4% following incorporation of AgNPs at a concentration of 5 mg/mL.The toxicity potential of the nanocomposite was also investigated. Nearly 80% cell viability was maintained in PMMA containing AgNPs at 10 mg/mL, indicating biocompatibility and low toxicity of the nanocomposite [122]. The evidence demonstrates that AgNP incorporation into dental formulations can inhibit cariogenic pathogens, improve biofilm resistance, and support tissue repair, highlighting their potential use across preventive, restorative, and regenerative dentistry

Beyond conventional dental materials and restorative approaches, AgNPs have also been investigated in broader oral healthcare settings. Recent studies have explored alternative delivery methods and biological strategies for preventing oral infections and controlling pathogenic biofilms. Praharsha *et al.* [123] developed an approach by coating bacteriophages with AgNPs. Antibacterial activity was evaluated using two oral pathogens, *S. mutans* and *Porphyromonas gingivalis*. The inhibition zone was measured as 14.50 mm for *S. mutans* and 16.83 mm for *P. gingivalis*. The positively charged AgNPs exhibited strong electrostatic adhesion to the negatively charged phages. Cytotoxicity testing on human gingival fibroblasts revealed 91% cell viability at 0.05% AgNPs after 24, 48, and 72 h, showing low cytotoxicity [123]. In another study, Tomiyama *et al.* assessed the effects of an AgNP-containing mouthrinse (NS-mouthrinse) on polymicrobial oral biofilms [124]. NS-mouthrinse significantly reduced bacterial cell viability and inhibited bacterial regrowth, as shown by a marked decrease in colony-forming units (CFU/mL) both immediately after treatment and following 48 h of regrowth. These findings indicated antimicrobial efficacy comparable to or greater than that of chlorhexidine gluconate, which is a commonly used oral antiseptic. In addition, the NS-mouthrinse reduced lactic acid production within the biofilm, reflecting reduced bacterial metabolic activity.

#### 3.2.3. Medical Device and Implant Coating Applications of AgNPs

AgNPs are used as coatings on the surfaces of medical devices and implants. This coating enhances the usability of the device and implant. In addition, it strengthens these devices from an antibacterial perspective [125].

Kim *et al.* tested the antibacterial effect of AgNPs in implantable medical devices. They synthesized AgNPs on polydimethylsiloxane, which is used in implantable medical devices [126]. The Ag^+^ obtained from AgNPs through oxidation had a destructive effect on bacteria. They damaged metabolic activities by producing ROS in bacterial cells. In addition, these ions threaten the life of the cell by binding to proteins in the cell. According to the results obtained in this effective study, the coating was found to be highly effective against both *E. coli* and *S. aureus* strains. Also, Ag^+^ obtained from AgNPs show high antibacterial activity, especially in implants that can be placed inside the body. De Giglio *et al.* are investigating the effect of AgNPs on titanium implant coatings [127]. AgNPs have multiple important effects on implants. AgNPs significantly reduce bacterial growth and biofilm formation on device surfaces. In addition, they increase the effect by reducing toxicity in this area during drug administration. In antibacterial effect tests, AgNPs have largely stopped the growth of multiple bacterial species. It is important that AgNPs, along with these implants, are compatible with the body. In this case, the effect of AgNPs within the body was also examined. As a result, thanks to the carefully adjusted amount of Ag^+^, it has become a center of benefit rather than harm. In the tests conducted, although AgNPs exerted some pressure on human osteoblast-like (bone) cells (MG63) in the first 24–48 h, it was observed that the cells regained their viability and exceeded 60% after 7 days. In short, this coating acts as a strong antibacterial barrier. Zhang *et al.* investigated the effect of AgNPs/Polytetrafluoroethylene (PTFE) hybrid coating on the surface of metallic implants [128]. The coating was formed layer by layer by depositing a polydopamine (PDA) substrate layer onto stainless steel, followed by a sol–gel matrix containing PTFE, and finally AgNPs. The importance of AgNPs in this coating is that they cause bacterial cell death by releasing Ag^+^. The biofilm-inhibiting effect of AgNPs has also been proven by reducing the adhesion of *E. coli* bacteria to the implant surface by 50–80%. In addition, this hybrid coating has reduced the corrosion current density by approximately tenfold. Therefore, the implant has become more resistant inside the body. Furthermore, its cytotoxicity was found to be high based on experiments conducted on mice. The study results have once again proven that AgNPs can have a strong coating structure and an effective antibacterial mechanism.

Overall, the evidence indicates that AgNP-based surface coatings provide dual benefits for medical implants: strong antibacterial activity through Ag ion release and ROS generation, and improved material durability and corrosion resistance. Importantly, these effects can be achieved with minimal long-term cytotoxicity when Ag levels are properly controlled.

#### 3.2.4. Systemic and Topical Anti-Infective Therapies

AgNPs can be used as a powerful and versatile tool against infectious diseases. They are effective against bacteria, fungi, and viruses. They have multiple modes of attack. They disrupt the bacterial cell wall and membrane integrity and destroy essential enzymes. In addition, they cause DNA and protein damage by generating ROS. They can also inhibit biofilm formation [129]. There are studies in the literature on the use of AgNPs as anti-infective tools. The study by Chhibber *et al.* [130] describes the topical application of AgNPs to burn wounds using a special carrier system called an emulgel. According to this study, they tested the use of histidine-coated AgNPs (H-AgNPs) to treat burn wound infections caused by *K. pneumoniae* bacteria. The efficacy of AgNPs as a topical anti-infective has been demonstrated *in vivo* and *in vitro*. This H-AgNPs emulgel application completely cleared the infection within 14 days. AgNPs also significantly reduced inflammatory markers in the body. Furthermore, no irritation was observed after 7 days of use on the skin. At the same time, antibacterial tests showed that the bacterial load in mice treated with H-AgNPs decreased by approximately 1000 times compared to those not treated with H-AgNPs. This study shows us that AgNPs can be highly active antibacterial agents in topical applications [130]. Shao *et al.* [131] examine the topical antibacterial efficacy and effects on wound healing of adding AgNPs to chitosan-based membranes. The study emphasizes that AgNPs play a critical role in preventing microbial infection in medical devices such as wound dressings and reducing dressing change frequency by slowly releasing Ag^+^ ions. Both *in vitro* and *in vivo* experiments have demonstrated that AgNPs provide strong antibacterial protection. However, due to the barrier formed by proteins in biological fluids, high AgNP loading is required to achieve real success in tissues. Consequently, this system can topically control infection without harming the wound healing process and tissue response [131]. In another study, Du *et al.* [132] tested the effect of AgNPs on sepsis, a systemic infection caused by the *E. coli* CQ10 strain, using mouse models. The AgNPs used in the study were coated with mice serum protein (MS-AgNPs). These applied mice serum made the AgNPs more resistant than AgNPs coated with PVP. Significant success was also observed in the treatment of sepsis. These MS-AgNPs reduced the mortality rate in mice infected with multidrug-resistant bacteria from 70% to 25%. This success was not observed with PVP-coated AgNPs. MS-AgNPs reduced the bacterial load in the blood and liver by approximately 100-fold within the first 8 h after infection. Based on this result, we can say that the spread of infection was prevented. These *in vivo* tests also showed no signs of serious toxicity in the liver and kidney functions of the mice. Ultimately, with the right coating, AgNPs can be both powerful antibacterial agents and effective against systemic infections such as sepsis [132].

#### 3.2.5. Diagnostic and Therapeutic Synergies

AgNPs offer diagnostic signal generation and traceability capabilities in addition to their potent antibacterial therapeutic effects, based on their optical and surface properties. The combination of these features creates a functional synergy between diagnostic and therapeutic processes, enabling real-time monitoring of treatment efficacy in infected areas [133].

Kim *et al.* [134] describe a hybrid NP system created by coating the surface of gold nanorods (AuNR) with a thin silver layer as a theranostic platform that combines the diagnostic and antibacterial functions of AgNPs. The silver coating initially suppresses the photoacoustic (PA) signal by masking the plasmonic properties of the gold core, but this coating eventually disappears as the silver layer oxidatively dissolves and releases Ag^+^ ions, allowing the PA signal of the gold core to appear again. The intensity of the recovered PA signal through this coating-dissolution process was found to be directly related to the amount of Ag^+^ ions released (R^2^ = 0.93–0.96), enabling real-time, non-invasive monitoring of the antibacterial dose at the infection site. As a result of the triggered Ag^+^ release, more than 99.99% of MRSA and *E. coli* bacteria were eliminated, and in the *in vivo* MRSA infection model, the bacterial load decreased by 1000-fold, accompanied by a 730% increase in the PA signal. These findings reveal that the silver coating is not only an antibacterial ion source but also a critical structural component that enables the controlled activation of the diagnostic signal [134]. Jain *et al.* [135] investigated the therapeutic antibacterial activity of AgNPs. The main objective of this study was to develop an S-gel formulation, a topical gel, for use in conditions such as burn and wound treatment. In the antibacterial studies conducted, AgNPs were effective against many bacterial strains tested. In addition, the success of the S-gel formulation, which is a hydrophilic gel, is highlighted. This gel was compared to a commercially available 1% silver sulfadiazine (SSD) cream. As a result of this comparison, it showed similar antibacterial effects despite containing 30 times less silver than SSD. They exhibited strong inhibition even after short-term interaction with bacteria. Accordingly, 10.5 h of inhibition was observed for *P. aeruginosa*. Testing a gel to be used therapeutically *in vivo* is also of vital importance. Accordingly, the safety of S-gel was tested by performing acute dermal toxicity tests on rats. According to the results, no adverse skin reactions were observed. Thus, AgNPs are potent agents that can be used therapeutically and as antibacterial agents [135].

AgNPs are effective in drug delivery systems. There are several studies in the literature.

A study conducted by Masri *et al.* [136] examined the importance of AgNPs in drug delivery and their effect on antibacterial activity. According to this study, Cephradine, an antibiotic, and Vildagliptin, an antidiabetic drug, were conjugated with AgNPs. The conjugation rate was determined to be 52% for Ceph-AgNPs and 62% for Vgt-AgNPs. Antibacterial tests also demonstrated that these conjugated drugs exhibited higher antibacterial activity than the drugs alone. For example, in *E. coli K1* bacteria, the use of Vgt-AgNPs reduced the bacterial count from 7.3 × 10^7^ to 5.4 × 10^5^ CFU. The antibacterial transport mechanism of these drugs is thought to be due to AgNPs attaching to the bacterial wall and penetrating the cell, thereby directly transporting the loaded drugs to the target bacteria. This comprehensive study demonstrates the effectiveness of AgNPs in drug transport and their antibacterial power [136]. Another study by Steckiewicz *et al.* [137] examined the role of AgNPs in the delivery of certain drugs effective in oral health and their antibacterial effect. According to this study, drugs such as CHX and metronidazole (MET) were delivered to the target area thanks to AgNPs. Two different delivery methods were used in this study. CHX was bound directly to the surface of AgNPs and transported. However, MET molecules were attached to the surface of AgNPs via linkers. Subsequently, the antibacterial efficacy of these conjugates against pathogens in the mouth was examined. Antibacterial tests showed that the combination of these drugs and AgNPs had a stronger antibacterial effect on some bacteria associated with periodontitis. MET alone required a concentration of more than 32 µg/mL to be effective against most bacteria. However, when bound to AgNPs, this concentration dropped to 2–16 µg/mL. This suggests that the delivery of drugs using AgNPs may enhance both the antibacterial activity and the activity of the drugs themselves [137]. In another study, Reddy *et al.* [138] evaluated the drug delivery properties and antibacterial efficacy of poly(acrylamide/sodium alginate)-based hydrogel composites (PASA/C30B/Ag) containing AgNPs and Cloisite−30B (C30B) clay were evaluated together. The addition of AgNP and clay altered the network structure of the hydrogel, reducing drug loading efficiency but still providing rapid delivery. Antibacterial tests showed that the AgNP-containing composites were significantly effective against both *Streptococcus faecalis* and *E. coli.* Specifically, the sample containing pure silver exhibited the strongest antibacterial effect with low MIC values. MIC values are 1.62 µg/mL for *S. faecalis* and 1.14 µg/mL for *E. coli.* Although a slight decrease in antibacterial activity was observed with increasing clay content due to partial coverage of the silver surface, all AgNP-containing systems are still much more effective than pure hydrogels. Overall, these results demonstrate that AgNPs impart a strong antibacterial character to the hydrogel system and that PASA/C30B/Ag composites are promising smart systems for drug delivery applications with high infection risk [138].

### 3.3. Wastewater Treatment Applications of AgNPs

AgNPs ability to inhibit proliferation of waterborne bacterial pathogens enable their use for the treatment of wastewater [139]. Yet, for AgNPs’ broad-term applicability in this field, their adverse impact on the environment must be addressed. Most of these adverse effects can be managed using green synthesis as it introduces a nature friendly and sustainable approach [139,140].

Polyethersulfone (PES) membranes containing green-synthesized AgNPs were investigated for their efficiency to purify wastewater in terms of salt rejection, water permeability, dye removal efficiency and antifouling potential. Synergistic activity between AgNPs and PES was shown by observable increase in the performance of the membrane. NaCl, CaCl_2_ and MgSO_4_ rejection increased to 57%, 41% and 67% from 32%, 27% and 26%, respectively, when AgNPs of 0.75% were loaded into the membrane. AgNP-PES membrane also effectively removed organic dyes including Congo Red and Methylene Blue (MB) and reduced the total fouling from 67% to 56% [141]. AgNP–coffee grounds waste (CWAg) nanocomposite was developed by Ghamdi *et al.* [142] to investigate their antibacterial activity and biosorption potential on the removal of MB and Cr (VI) from wastewater. CWAg effectively inhibited proliferation of *S. mutans*, MRSA and *Serratia marcescens* with inhibition zones of 26 mm, 18.66 mm and 21, while sole coffee grounds waste showed slight activity only against *P. aeruginosa*. CWAg nanocomposite effectively removed both MB and Cr (VI) with equilibration times of 120 and 90 min and half absorption times of 13.9 and 9.2 min, respectively [142]. AgNP-incorporated membranes were investigated for their ability to treat microorganism-rich seawater. Membranes led to complete bacterial inhibition (100%) approximately after 3 h for *E. coli* and *Vibrio coralliilyticus*, and 48 h for *S. aureus* and 72 h for *Exiguobacterium aestuarii*, where the longer inhibition times attributed to structural difference in Gram-positive bacteria. In tests with natural seawater from Sentosa Island, the reduction curve was slower due to microbial diversity. It was concluded that AgNP-enriched membranes can be utilized in seawater environments as effective bacterial barriers [143].

Overall, the discussed studies highlight that AgNP-enabled systems can simultaneously inhibit bacterial contamination and increase purification efficiency, whether through nanocomposite adsorption, improved membrane rejection, or seawater filtration.

## 4. Toxicity and Potential of AgNPs

**Figure 3:**
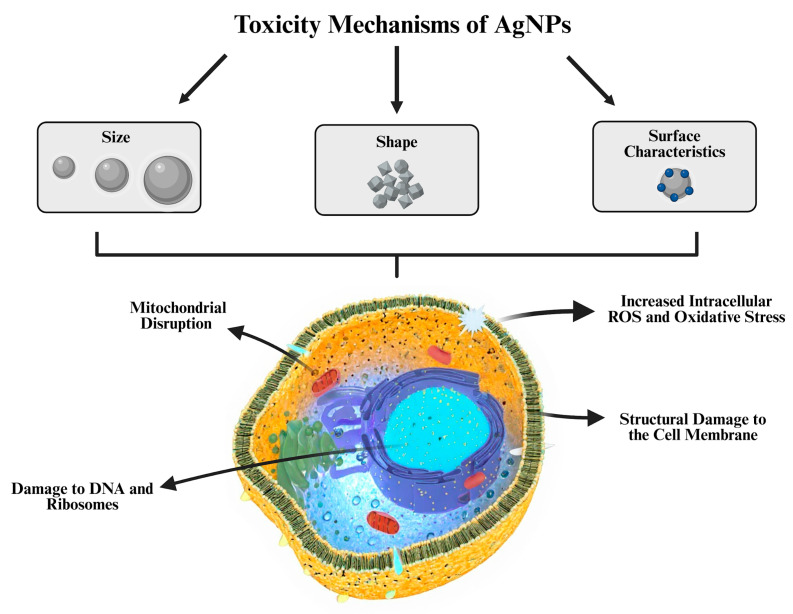
Toxicity Mechanisms of AgNPs. The toxicity profile of AgNPs is dependent on size, shape, and surface characteristics, as depicted in the upper boxes. These properties influence the interaction with the cell, leading to structural damage to the cell membrane and organelles. Directional arrows illustrate the downstream toxic effects, specifically highlighting mitochondrial disruption, ROS generation, and damage to DNA and ribosomes [23].

AgNPs have emerged as widely utilized nanomaterials, whether utilized alone or alongside functional molecules. Their applications extend across agriculture, antibacterial coatings, wound healing, dentistry, and water treatment, highlighting their wide-ranging potential [144]. However, their possible harmful effects need to be examined carefully, as wider use has raised concerns about their toxicity to people and the environment [145]. The toxicity of AgNPs is influenced by several factors, including stability, dose of administration and surface chemistry, which influence the aggregation of the particles in the organism. The toxicity potential is also affected by the physicochemical properties of AgNPs (Figure 3). Furthermore, AgNP-induced toxicity can be observed through various mechanisms, which differ based on the biological system. Key mechanisms include cell membrane disruption and excessive ROS generation, impairing organelle functions [146]. The toxicological profile of AgNPs has been investigated in both *in vivo* and *in vitro* studies [147].

Dinç *et al.* [148] investigated the toxicity potential of AgNPs in biological systems in a dose-dependent manner. The effects of AgNPs at concentrations of 10, 30, and 50 µg/mL were investigated on *Caenorhabditis elegans*, human vascular endothelial cells (HUVECs) and bacteria *E. coli* and *B. subtilis*. The initial evaluation in *C. elegans* revealed that AgNPs markedly affected both reproductive function and locomotion. At 10 µg/mL, the reproductive rate decreased by 25%. Following 6 days of exposure, body-bending frequency dropped from 42 to 19 beats. The results highlight the possible neurotoxic effect of AgNPs against *C. elegans*, with increased bioaccumulation. At 50 µg/mL, >90% of HUVECs lost viability, while lowered concentrations (10 µg/mL) resulted in a 25% reduction in viability within 48 h. The dose-dependent inhibition of bacterial growth was determined with 52% inhibition at 50 µg/mL and 23% at 10 µg/mL concentration [148]. Okuthe *et al.* [149] examined the acute dermotoxicity of green synthesized AgNPs in the epidermis of *Danio rerio*. Zebrafish were exposed to 0.031, 0.250, and 5000 µg/L of AgNPs for 96 h. No mortality was recorded, but clear signs of toxicity were observed. TEarly responses included rapid mucus production within the first 2 h, indicating acute stress. In controls, the cup cell count was 11.17 ± 3.148, and the average size was 12.71 ± 2.084 µm after 24 h. At 0.031 µg/L, cup cell count increased to 20.00 ± 2.275 after 24 h. Cell size decreased to 7.07 ± 1.22 µm at 24 h, increased to 15.85 ± 2.369 µm at 48 h, and dropped to 10.83 ± 1.339 µm after 96 h. At 0.250 µg/L, cell size reached 16.15 ± 2.298 µm at 48 h, with cup cell counts increasing to 18.94 ± 2.287 at 24 h and 18.89 ± 2.298 at 48 h. At 5000 µg/L, size decreased to 8.256 ± 1.382 µm and further to 6.403 ± 1.583 µm after 96 h, while cup cell count dropped significantly to 9.556 ± 1.423 at 48 h. These data indicate the onset of severe cellular damage and loss of epidermal regenerative capacity. Pathological epidermal lesions were observed in all treated groups, with the most severe effects at 5000 µg/L after 96 h. These findings show that green synthesis does not eliminate the toxic potential of AgNPs [149]. Matras *et al.* [150] investigated the phytotoxic effects of AgNPs with different surface properties and charges on monocotyledonous and dicotyledonous model plants. Three types of AgNPs were synthesized using trisodium citrate (TCSB-AgNPs), tannic acid (TA-AgNPs), and cysteamine hydrochloride (CHSB-AgNPs). CHSB-AgNPs exhibited a positive surface charge, whereas TCSB-AgNPs and TA-AgNPs carried negative charges. The strongest phytotoxic effect was observed in positively charged CHSB-AgNPs [150]. Cunningham *et al.* [151] investigated the size-dependent toxicity of AgNPs. The AgNPs were coated with a hybrid lipid membrane to prevent surface oxidation and Ag^+^ release. In general, the study was based on the application of AgNPs ranging in size from 20 to 100 nm at concentrations of 0.25 to 12 mg/L to zebrafish for 5 days. The first observed toxicity effect was mortality. In general, an increase in mortality was observed with an increase in AgNP concentration and a decrease in AgNP size. Specifically, 100% mortality was observed in AgNPs with a size of 20 nm and a concentration of 12 mg/L. In those between 20 and 40 nm, more than 75% of mortality occurred at 24 h post-fertilization. In addition, no significant mortality occurred in NP particles ranging from 80 to 100 nm. Another toxic effect is malformations in fish. The findings showed that AgNPs with average size of 100 nm exhibited reduced internalization, highlighting the inverse relationship between the toxicity and particle size [151]. Such studies have highlighted the potential toxic effects of AgNPs across biological systems, with dose, size, and surface characteristics emerging as critical determinants. Dinç *et al.* [148] reported significant reproductive, locomotor, and viability reductions in *C. elegans* and HUVECs, demonstrating dose-dependent toxicity, while Okuthe *et al.* [149] found clear dermal stress responses and epidermal damage in Danio rerio, even at low concentrations. Toxicity was also evident in plants, where Matras *et al.* [150] showed that positively charged AgNPs caused stronger phytotoxic effects than negatively charged particles. Complementing these results, Cunningham *et al.* [151] found that smaller AgNPs caused higher mortality and anatomical malformations in zebrafish embryos, reinforcing that higher doses and smaller particle sizes increase toxicity risk. Together, these studies demonstrate that AgNPs can have harmful effects depending on concentration, exposure duration, particle size, and surface chemistry.

In another study, Ayubee *et al.* [152] highlighted the toxicity of AgNPs and AgNP–ampicillin conjugates on the Vero cell line. The cells were treated with 25 µL of AgNP combinations at a concentration of 100 µg/mL for 24 h. Results revealed cytotoxic effects of AgNPs, resulting in approximately 80-90% of cell viability. However, AgNP-ampicilin conjugates notably decreased these effects, achieving a cellular viability of greater than 95%. Researchers attributed this lower toxicity to the shielding effect of the ampicillin, which may prevent direct interaction of AgNPs with mammalian cell membranes. Additionally, they stated that the presence of silica coating and amine functionalization during synthesis can further help to improve the biocompatibility of the conjugates [152]. Das *et al.* [153] evaluated the toxicity potential of chemically synthesized and AgNPs synthesized using Phe-Gly peptide-gallol conjugate on mouse embryonic fibroblast cell lines. It was shown that peptide–gallol capped AgNPs had reduced toxic effects, achieving almost a two-fold reduction compared to chemically synthesized AgNPs. The IC_50_ values were reported as 250 μg/mL for peptide–gallol capped AgNPs, while chemically synthesized counterparts had an IC_50_ of 100 μg/mL. The observed reduction in toxic effects was associated with free phenolic -OH groups in the peptide-gallol moiety, which may scavenge ROS and thereby mitigate oxidative damage in cells [153]. Mortazavi Moghadam *et al.* [154] investigated the toxicity potential of AgNPs in food coatings when ingested through packaging materials. Rainbow trout (*Oncorhynchus mykiss*) fillets were used during the experimentation considering their proneness to spoilage. To reduce AgNP migration, a composite matrix consisting of cellulose and low-density polyethylene (LDPE) was developed. Fish fillets contained 1.85 μg/mL AgNPs, and *in vivo* studies in Wistar rats were conducted to assess its potential impact. There was no observable AgNP accumulation detected in the liver, kidneys, small intestine, or blood. The study further examined how migrated AgNPs affect the oral microbiota, focusing on *Streptococcus mutans* despite the oral cavity’s diverse microbial community. According to MIC results, 0.5 mg/mL nanosilver was sufficient to inhibit *S. mutans*. In comparison, ICP-MS measurements revealed that the packaging released a maximum of 1.85 μg/mL AgNPs. This amount was insufficient to affect *S. mutans* or alter oral microbiota. The *in vivo* results indicate that AgNP migration from the cellulose/LDPE nanocomposite is minimal and insufficient to cause detectable biological effects [154].

In contrast, several investigations have shown that AgNP–related toxicity can be mitigated through controlled formulations or surface modifications. Ayubee *et al.* [152] demonstrated that coupling AgNPs with ampicillin reduced cytotoxic effects in mammalian cells, likely due to decreased direct contact with cell membranes, while Das *et al.* [153] showed that peptide-gallol capped AgNPs caused roughly half the toxicity of chemically synthesized NPs, attributed to ROS-scavenging phenolic groups. Real-world exposure experiments also suggest limited risk, as Mortazavi Moghadam *et al.* [154] found minimal AgNP migration from cellulose/LDPE food packaging and no detectable accumulation in rat organs. Collectively, these studies highlight that although AgNPs possess inherent toxic potential, safer design strategies, such as conjugation, surface coating, and dose control, can substantially reduce biological impact and support safer implementation.

AgNP toxicity is extensively examined in the literature and is influenced by various factors, including the synthesis method. Among available approaches, green synthesis is now widely preferred, as it offers reduced environmental impact relative to chemical and physical synthesis methods [155]. Additionally, green-synthesized AgNPs are valued for their sustainability and biocompatibility. Unlike chemical methods, green synthesis uses biological entities such as plants as both reducing and stabilizing agents. In this context, Farhadi *et al.* [156] conducted a comparative analysis of the toxicity potential of AgNPs synthesized using three different routes, including chemically-synthesized (c-AgNPs), green-synthesized from *Spirulina* extracts (g-AgNPs) and green-synthesized and capped with chitosan (CH-gAgNPs). Cell viability assays performed on the H9c2 cell line demonstrated the promotion of the healthy cells on samples treated with g-AgNPs compared with c-AgNPs. CH-gAgNPs further reduced the cytotoxicity against healthy cells, exhibiting no detectable toxicity among all tested concentrations ranging from 10 to 100 µg/mL. In addition, the authors emphasized the immense antibacterial potential of CH-gAgNPs against both Gram-positive and Gram-negative bacterial strains, suggesting their potential as a safe and biocompatible alternative to address the toxicity issues associated with conventional AgNPs [156].

Other factors, including concentration, pH, and synthesis conditions, also influence physicochemical properties and stability. AgNPs obtained from plants can be derived from leaf extracts, seed extracts, aerial parts, and whole plant extracts. Furthermore, phytochemicals such as terpenoids, alkaloids, and polyphenols play an important role in plant-based syntheses. For example, terpenoids interact with Ag^+^ due to the functional groups in their structures and stabilize the NPs. Furthermore, alkaloids provide electrons to reduce Ag^+^ ions. In synthesis using algae, proteins and polysaccharides found in algae are used as reducing agents. Additionally, various bioactive organic compounds in their structures and functional groups, such as hydroxyl and carboxyl groups, help reduce Ag^+^ ions. In synthesis using fungi, silver ions are reduced and stabilized simultaneously by utilizing extracellular proteins and enzymes. In bacteria-based synthesis, the size advantage is exploited. Bacteria are preferred due to their small size and their use of the two most basic reducing enzymes, reductases and dehydrogenases, found in their structures. Their use in controlled synthesis is quite high [155,157].

Synthesis using traditional methods has various advantages, such as precise structural control and obtaining high particle yields [158]. However, usage of reducing and stabilizing chemicals during AgNP production introduces significant environmental and health risks, along with high costs and energy-intensive processes [159]. On the other hand, green synthesis offers sustainable, biocompatible, and environmentally friendly alternatives such as plants, algae, fungi and bacteria [158]. It reduces environmental impact through the use of non-toxic reducing and stabilizing agents while providing cost-effective and manageable processing steps [160]. Still, green synthesis faces challenges such as variability, slower reaction rates and scalability. Further research into optimizing green synthesis methods is crucial for reducing environmental impact and ensuring scalability [158].

## 5. Future & Conclusions

AgNPs demonstrate high antibacterial efficiency and possess synergistic effects when applied in combination with antibiotics. AgNPs produced via different synthesis methods have been widely tested against different bacterial strains and showed efficiency even towards the drug-resistant ones. Based on the current literature, it is reported that AgNPs exert their bactericidal effects using different mechanisms. Among these, bacterial membrane disruption and ROS generation are the widely recognized ones where both contribute to inhibition of the bacterial proliferation. In addition, AgNPs can interfere with cellular components like ribosomes and DNA and enhance their antibacterial effects

AgNPs physicochemical properties including size, shape and surface charge also influence their antibacterial activity. For example, smaller sized AgNPs show increased bactericidal effects as they can penetrate more easily to bacterial membranes compared to their larger counterparts. AgNP morphology is another factor affecting the antibacterial activity as indicated in the literature by spherical AgNPs achieving more potent effects than triangular and disc-shaped ones. Surface charge of AgNPs also contributes to their antibacterial potential by influencing the electrostatic interactions between NP and the bacterial membrane. Surface functionalization has also gained importance as it is employed to improve biocompatibility, stability and targeted activity of AgNPs.

Remarkable antibacterial potential of AgNPs extend their application across numerous fields. Agriculture is one of these fields where AgNPs are highly utilized as nanopesticides, nanofertilizers and growth-promoting agents based upon their positive impact on germination, plant growth and yield. Food packaging and preservation also represent important agricultural applications where researchers develop AgNP-incorporated packaging films or solutions to extend shelf life while preventing spoilage of the products. In addition, AgNP coatings are widely applied as they are able to indicate food spoilage through temperature- and pH-sensitive changes to provide real-time information about food quality to consumers. Additionally, utilization of AgNPs in the field of agriculture extends to the development of functional textiles with increased antibacterial potential.

Beyond agriculture, the biomedical field is another major area where AgNPs are broadly used. For instance, extensive *in vitro* and *in vivo* research has shown the potential of AgNPs for developing wound healing materials. Currently, AgNP-incorporated hydrogels are produced to lower infection through their antibacterial potential and consequently providing an accelerated healing process. AgNPs are also applied as coatings for biomedical devices to reduce the likelihood of infection. However, their application in dentistry remains limited and requires further research.

The toxicity potential of AgNPs remains a major issue even though they are increasingly utilized in a range of applications. Chemically synthesized AgNPs may pose risks to human health and the environment. Although many studies have been conducted, safe usage practices remain insufficient. Accordingly, recent studies have emphasized the development of rational design and application strategies to mitigate these toxicity concerns and support the safe clinical translation of AgNP-based antibacterial systems. In this context, surface functionalization and coating approaches have gained increasing attention, as they have been shown to improve AgNP stability and biocompatibility while limiting uncontrolled silver ion release and direct NP-cell interactions. Notably, conjugation of AgNPs with conventional antibiotics, bioactive polymers, or peptide-based ligands has emerged as a promising strategy, as these systems can preserve antibacterial efficacy while reducing AgNP-associated cytotoxicity through effective dose minimization and shielding effects. Collectively, these formulation-based strategies highlight feasible pathways for improving the safety profile of AgNP-based antibacterial platforms and facilitating their translational advancement.

Beyond post-synthesis formulation approaches designed to mitigate AgNP-associated toxicity, the future of AgNP applications is expected to rely on comprehensive toxicity assessments and the development of clear safety guidelines. In this context, green synthesis offers an alternative approach to reducing the toxicity associated with chemically synthesized AgNPs. It is particularly attractive due to its low cost, improved biocompatibility, and sustainability Ultimately, green-synthesized AgNPs can reduce the reliance on hazardous chemicals associated with toxicity.

In conclusion, AgNPs demonstrate high potential with their significant antibacterial potential and wide range of applicability. Nevertheless, achieving safer applications will depend on improved synthesis strategies and a more comprehensive understanding of their underlying toxicity mechanisms. Emphasizing green synthesis and safety evaluations for AgNPs will facilitate their effective and sustainable application across diverse applications, mainly in antibacterial research.

## Figures and Tables

**Table 1:** Recent Antibacterial Applications of AgNPs.

Application Type	AgNP Type	Physicochemical Property	Main Results	References
Antibacterial (*in vitro*)	Green synthesized from *Hyoscyamus muticus* extract	Globular morphology 17.3 ± 4.5 nm size Zeta potential of −64.42 mV SPR value 398 nm	Inhibition zone of 22 mm against *S. aureus*. Inhibition zone of 19 mm against *A. baumannii.*	[15]
Antibacterial (*in vitro*)	Green synthesized from *Bacillus vietnamensis* JA01	Spherical morphology Size ranging from 14–21 nm SPR value of 381.50 nm	Strong antibacterial activity against several pathogenic bacterial strains. Inhibition zones ranging from 0.3 ± 0.2 nm to 1.8 ± 0.2 nm.	[81]
Antibacterial (*in vitro*)	Green synthesized from probiotic bacterial strain *Lacticaseibacillus rhamnosus*	Globular morphology Average size of 199.7 nm SPR value of 443 nm Zeta potential value of −36.3 mV	Significant antibacterial activity against various bacterial strains. Inhibition zone of 16 mm against *Vibrio parahaemolyticus*.	[82]
Antibacterial (*in vitro*)	Green synthesized from *Cocos nucifera* extract	Globular morphology 28 nm size SPR value 425 nm	Significant inhibitory activity against tyrosinase. Influence on skin rejuvenation. Strong antibacterial activity against pathogens related to skin infection.	[83]
Antibacterial (*in vitro*)	Green synthesized from *Momordica charantia* extracts	Predominantly spherical morphology Size ranging from 14.5 ± 8.2 to 70 nm The SPR value between 420–450 nm	Strong antibacterial activity against *E. coli* ATCC25922. AgNPs between 0.625 and 2.50 mg/L achieved 99.99% killing after 24 h of incubation.	[84]
Agricultural	Green synthesized from *Dianthus superbus* L. extract	Globular and polydisperse morphology Size between 11–18 nm	AgNPs prevented discoloration, decay, and spoilage of grapes for 4 days. Extended shelf life of grapes.	[85]
Agricultural	Green synthesized from *Thelypteris erubescens*	Globular morphology Sizes ranging from 30 to 40 nm.	Strong antibacterial activity against several bacterial strains. Dose-dependent increase in dry weight, germination rate, and crop yield in maize.	[86]
Agricultural	Green synthesized from *Strobilanthes crispus*	Globular morphology Average size of 75.25 nm Zeta potential of −35.6 mV	Strong antibacterial activity against *P. aeruginosa* and *Streptococcus mutans.* Disruption of gene expression responsible for bacterial adhesion and biofilm formation.	[87]
Agricultural	Green synthesized from the fungus *Phoma herbarum*	Spherical morphology Size ranging from 4 to 40 nm SPR peak around 429 nm	Dose-dependent antifungal activity against onion basal root rot disease causing *Fusarium oxysporum f.sp. cepae.* Inhibition rate of 94.42% *in vivo*.	[88]
Agricultural	Green synthesized from spent mushroom substrate	Globular morphology Zeta potential of −48.3 ± 0.58 mV	Dose-dependent antibacterial activity against several bacterial strains through protein and amino acid leakage mechanisms. Minimal cytotoxicity against human dermal fibroblasts.	[89]
Wound Healing	Green synthesized from taro corms extract	Spherical morphology Size between 244.9 and 272.2 nm SPR values between 438–445 nm Zeta potential of −18.8 mV	Complete wound closure in rabbits following 14 days. Increase in collagen content. Strong antibacterial activity against several bacterial strains.	[90]
Wound Healing	Green synthesized from macrofungus *Phellinus adamantinus*	Globular morphology Size ranging between 40–50 nm	Bactericidal activity against several bacterial species. 20% wound healing efficacy following 24 h of treatment.	[91]
Wound Healing	Green synthesized from *Ocimum sanctum*	Globular morphology 28.95 ± 7.74 nm in size Zeta potential of −17.8 mV	45% healing of scratch wounds in 12 h *in vitro.* Reduced toxicity against L929 mouse fibroblasts. Strong antibacterial activity against *E. coli.*	[92]
Wound Healing	Green synthesized from *Tribulus terrestris*	Spherical morphology Size of 50.2 nm SPR peak at 410 nm	Antibacterial and anti-inflammatory effect in wounds infected with *P. aeruginosa* Faster and more pronounced histological development through administration of AgNPs Low toxicity	[93]
Wound Healing	AgNPs were commercially purchased	Size of 5 nm SPR peak at 400 nm	Notable antibacterial activity against infected skin defects. Reduced toxicity. Promotion of angiogenesis and acceleration of wound closure.	[94]
Dental	Synthesized via chemical reduction	Hemispherical and irregular morphology Size between 2–17 nm	AgNPs added to the PMMA surface strongly inhibit biofilm formation of *S. aureus.*	[95]
Dental	Synthesized via chemical reduction	Spherical morphology Size ranging from 5.2 ± 1.2 nm to 37.4 ± 3.6 nm Zeta potential ranging from −48.4 ± 6.9 mV to −52.6 ± 8.5 mV SPR value between 408–410 nm	Significant potential for preventing dental caries. Strong antimicrobial activity in several sizes.	[96]
Dental	Synthesized via chemical reduction	Globular morphology Size ranging from 10.2 ± 0.7 nm to 29.3 ± 2.3 nm Zeta potential between −35.0 ± 3.3 mV to −52.6 ± 8.5 mV	Size and oral biofilm dependent antibacterial activity. High resistance in oral biofilms obtained from disabled patients against AgNP.	[97]
Dental	Synthesized using green synthesis and chemical synthesis	Globular morhpology Size between 2–20 nm SPR peak at 400 nm	Significant antibiofilm inhibition. Notable biocompatibility. Concentration-dependent toxicity.	[98]
Dental	Synthesized via chemical reduction	Spherical morphology -Size between 10–100 nm -Zeta potential value −24.75 mV	Strong antibacterial effect against various bacteria in synergy with Ebselen, a synthetic organoselenium drug molecule. Excellent cell compatibility. Strong anti-inflammatory activity. Reduced alveolar bone resorption in rats with periodontitis.	[99]
Water Treatment	NPs were commercially purchased	Size between 2–5 nm	Inhibits biological nitrogen removal. Nitrification is suppressed by up to 90%. Reduces the potential of bacteria playing a key role in the nitrogen cycle in water.	[100]
Water Treatment	Green synthesized from *Cynometra ramiflora* leaf extract	Spherical morphology Average size of 18.84 ± 8.67 nm Zeta potential of −18.2 mV SPR value peaks at 431 nm	Strong catalytic effect on methyl orange in water purification. 95% degradation of methyl orange in 15 min. Rapid and effective degradation of azo dyes in water.	[101]
Water Treatment	Green synthesized from *Melia azedarach*	Globular morphology Average size of 13.6 nm zeta potential of +50 mV	Microbial contaminants in hospital wastewater were removed using cold plasma and AgNPs. Complete colony removal was observed.	[102]
Water Treatment	Green synthesized from cell-free supernatant of *Actinomycetes*	Spherical morphology Size ranging from 5 nm to 45 nm	Strong antibacterial activity against various pathogens. 100% bacterial elimination.	[103]
Water Treatment	Green synthesized from *Trigonella foenum-graecum* aqueous extract	Spherical morphology Size between 20–50 nm SPR peak at 439.29 nm	Separated 94.5% of crystal violet dye from the medium. Strong antibacterial activity against various bacterial strains.	[104]

## Data Availability

No new data were created or analyzed in this study. Data sharing is not applicable to this article.

## Abbreviations

The following abbreviations are used in this manuscript:

AgNPs	Silver nanoparticles
NPs	Nanoparticles
MIC	Minimum inhibitory concentration
MBC	Minimum bactericidal concentration
ROS	Reactive oxygen species
DNA	Deoxyribonucleic acid
MDR	Multidrug-resistant
MRSA	Methicillin-resistant *Staphylococcus aureus*
IC_50_	IC_50_ is defined as the concentration of a drug required for 50% inhibition of biological or biochemical function
CFU	Colony-forming units
PEI	Polyethyleneimine
PpIX	Protoporphyrin IX
Cfx-AgNPs	Ceftriaxone-conjugated silver nanoparticles
DMT	4-amino−3,5-dimercapto−1,2,4-triazole
SPR	Surface plasmon resonance
PMMA	Polymethyl methacrylate
PVP	Polyvinylpyrrolidone
CHX	Chlorhexidine
EFB	Empty fruit bunches
H-AgNPs	Histidine-coated silver nanoparticles
HUVEC	Human vascular endothelial cells
